# Improved Bioavailability of Stilbenes from *Cajanus cajan* (L.) Millsp. Leaves Achieved by Hydroxypropyl-*β*-Cyclodextrin Inclusion: Preparation, Characterization and Pharmacokinetic Assessment

**DOI:** 10.3390/molecules30122526

**Published:** 2025-06-10

**Authors:** Yingya Qiu, Jiangxuan Lai, Yuhan Zhang, Sheng Fang, Zili Guo, Xianrui Liang

**Affiliations:** 1 Key Laboratory for Green Pharmaceutical Technologies and Related Equipment of Ministry of Education, College of Pharmaceutical Sciences, Zhejiang University of Technology, Hangzhou 310014, China; qiuyingya0326@163.com (Y.Q.); laijianngxuan@163.com (J.L.); zhangyuhan2001619@163.com (Y.Z.); 2 School of Food Science and Biotechnology, Zhejiang Gongshang University, Hangzhou 310018, China; fangsheng@zjgsu.edu.cn; 3 Key Laboratory of Pollution Exposure and Health Intervention of Zhejiang Province, Zhejiang Collaborative Innovation Center for Full-Process Monitoring and Green Governance of Emerging Contaminants, Interdisciplinary Research Academy, Zhejiang Shuren University, Hangzhou 310015, China

**Keywords:** stilbenes, *Cajanus cajan* (L.) Millsp. leaves, 2-hydroxypropyl-*β*-cyclodextrin, inclusion complex, bioavailability

## Abstract

Despite their broad pharmacological potential, the clinical application of stilbenes derived from *Cajanus cajan* (L.) Millsp. leaves (CCMLs) is limited by their poor aqueous solubility, resulting in low oral bioavailability. In this study, an inclusion complex of stilbenes extracted from CCMLs and 2-hydroxypropyl-*β*-cyclodextrin (HP-*β*-CD) was successfully developed to improve their solubility and oral bioavailability. The preparation conditions were optimized using the response surface methodology, with an optimum mass ratio of CCML extract (CCMLE) to HP-*β*-CD of 1.0:8.2 (g/g) and an optimal temperature of 33 °C for 2 h. The maximum inclusion efficiency of stilbenes was 82%, and the physicochemical properties of the inclusion complex were characterized. Both in vitro dissolution studies and in vivo pharmacokinetic evaluation demonstrated that HP-*β*-CD encapsulation significantly improved the solubility and dissolution rate of stilbenes compared to those of unformulated CCMLE. Notably, the relative bioavailability of cajaninstilbene acid (CSA), longistyle C (LLC) and 4-*O*-methylpinosylvic acid (MPA) increased to 198%, 177% and 131%, respectively. This work provides a simple and effective strategy to enhance the solubility and bioavailability of naturally derived stilbenes, offering promising potential for the development of other insoluble natural components for both functional food and pharmaceuticals.

## 1. Introduction

Stilbenes are an important class of plant-derived secondary metabolites characterized by a 1,2-diphenylethylene skeleton. They are synthesized via the phenylpropanoid/polyketide pathway in response to biotic or abiotic stress. In recent years, stilbenes have attracted considerable interest in both the pharmaceutical and food industries due to their diverse bioactivities, including anticancer, neuroprotective, anti-diabetic and anti-inflammatory effects [1,2]. Despite their promising bioactivities, stilbenes are found predominantly in a limited number of plants, including Dipterocarpaceae, Fabaceae and Vitaceae [3,4,5,6].

**Figure 1:**
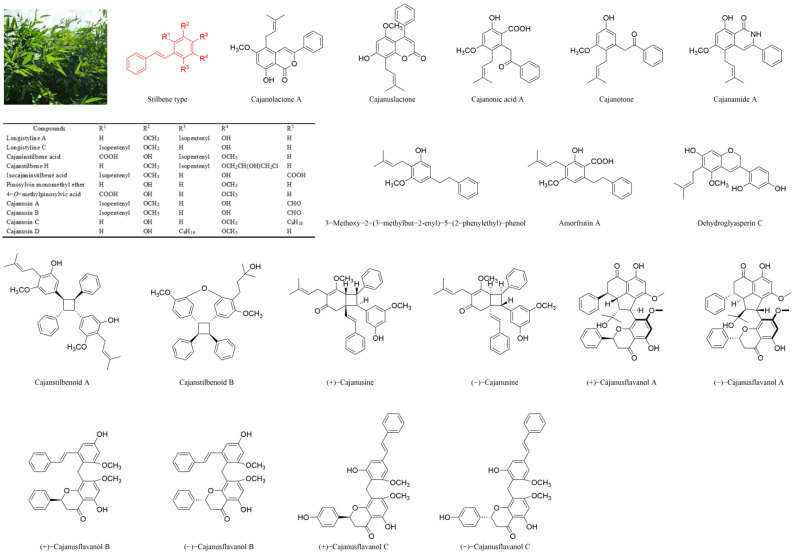
Structures of parts of stilbenes from *Cajanus cajan* (L.) Millsp. leaves (CCMLs).

*Cajanus cajan* (L.) Millsp., commonly known as pigeonpea, is a leguminous Fabaceae plant widely cultivated in Asia, Africa and South America [7,8]. Dried *Cajanus cajan* (L.) Millsp leaves (CCMLs) are abundant in stilbenes, flavones and other bioactive ingredients and have been used in traditional Chinese medicine for the treatment of femoral head necrosis [9,10], osteoporosis [11,12], hypertension [13] and diabetes [14]. To date, more than 30 distinct stilbenes have been identified in CCMLs (Figure 1). Among them, cajaninstilbene acid (CSA), longistyle C (LLC) and 4-*O*-methylpinosylvic acid (MPA) are the major active compounds, which are employed for the management of various conditions including malignancies, Alzheimer’s disease (AD), depressive disorders and ischemic stroke [15]. Specifically, stilbenes such as CSA have been shown to exert neuroprotective effects against AD by reducing the generation and oligomerisation of amyloid-*β* (A*β*), enhancing its clearance and inhibiting tau hyperphosphorylation and aggregation. They also mitigate A*β*-induced neurotoxicity and neuroinflammation by targeting microglia and astrocytes [15,16]. These compounds act as potent antioxidants, scavenging reactive oxygen species (ROS), and activate the SIRT1 pathway, which modulates neuronal survival by deacetylating key substrates such as p53 and PGC-1α. This offers multi-target therapeutic potential for the treatment of AD [17]. Furthermore, CSA promotes PC12 cell survival, reduces lactate dehydrogenase (LDH) leakage and suppresses corticosterone-induced apoptosis and DNA damage, exhibiting antidepressant properties [18]. LLC exhibits potent antimicrobial activity against methicillin-resistant Staphylococcus aureus (MRSA) by inhibiting biofilm formation and suppressing the expression of MRSA virulence genes [19]. MPA modulates the PI3K-AKT, insulin, FOXO and AGE-RAGE signalling pathways, contributing to its hypoglycaemic effects and its potential to treat metabolic disorders [20].

Despite their broad pharmacological potential, the clinical application of CCML-derived stilbenes is limited by their poor aqueous solubility, resulting in low oral bioavailability. As inadequate bioavailability compromises therapeutic efficacy assessment, strategies to improve the solubility and bioavailability of CCML-derived stilbenes are crucial for their further development as functional food ingredients and therapeutic agents.

In recent years, nanoencapsulation has attracted considerable attention as a promising strategy for improving the delivery of poorly water-soluble bioactive compounds. Systems using nanoencapsulation exhibit excellent biodegradability, improved stability, controlled release, reduced toxicity, enhanced aqueous solubility and increased bioavailability and efficacy [21,22]. Cyclodextrins are cyclic oligosaccharides composed of α-1,4-linked glucose units. As an FDA-approved carrier, cyclodextrins have superior biocompatibility and are widely used to enhance the solubility and bioavailability of hydrophobic, insoluble natural products by forming cyclodextrin inclusion complexes [23,24]. Our previous studies have also demonstrated that inclusion complexation by *β*-cyclodextrin (*β*-CD) significantly improved the solubitly and bioavailability of isoflavones extracted from red clover and chickpeas [25,26]. Among these carriers, 2-hydroxypropyl-beta-cyclodextrin (HP-*β*-CD), a chemically modified derivative of *β*-cyclodextrin, has emerged as a highly effective solubilizing excipient [27]. Structurally, HP-*β*-CD retains the conical oligosaccharide shape of *β*-CD with a hydrophilic outer surface and a hydrophobic inner cavity, allowing for the formation of non-covalent inclusion complexes with hydrophobic guest molecules. Compared to *β*-CD, HP-*β*-CD offers significantly higher aqueous solubility, reduced crystallinity and an improved safety profile, making it more suitable for pharmaceutical and nutraceutical applications [28,29,30]. Many studies have confirmed its effectiveness in enhancing the solubility, stability and bioavailability of various poorly soluble bioactive compounds through inclusion complexation [31,32].

Based on the superior complexing ability of HP-*β*-CD, a stilbene/HP-*β*-CD inclusion complex was prepared by encapsulating the poorly soluble stilbenes in *Cajanus cajan* (L.) Millsp. leaf extract (CCMLE) to form stilbene/HP-*β*-CD. Key preparation parameters, including the mass ratio of CCMLE to HP-*β*-CD, temperature, stirring speed (rpm) and reaction time were optimized. The prepared inclusion complex was characterized by ultraviolet–visible (UV-vis) spectroscopy, Fourier transform infrared (FTIR) spectroscopy and scanning electron microscopy (SEM). In vitro dissolution and in vivo pharmacokinetic studies were conducted to evaluate the performance of the stilbene/HP-*β*-CD inclusion complex. This was the first study to use HP-*β*-CD to enhance the solubility and bioavailability of CCML-derived stilbenes. It provides novel pharmacokinetic and tissue distribution data, demonstrating significant improvements in bioavailability and enhanced tissue penetration for CSA, LLC and MPA.

## 2. Results and Discussion

### 2.1. Preparation of Stilbene/HP-β-CD Inclusion Complex

#### 2.1.1. Optimization of Inclusion Complex Through Single-Factor Experiments

The inclusion complex of stilbenes in CCMLE and HP-*β*-CD was prepared and optimized through single-factor experiments. Four key parameters were evaluated for their effect on the encapsulation efficiency: the mass ratio of CCMLE to HP-*β*-CD, temperature, stirring speed (rpm) and reaction time (Supplementary Figure S1).

The mass ratio of CCMLE to HP-*β*-CD played a decisive role in determining the encapsulation efficiency. A ratio of 1:7 yielded the optimal encapsulation efficiency. Further increases in HP-*β*-CD led to a decrease in the encapsulation efficiency, probably due to the self-aggregation of excess cyclodextrin, which can sterically hinder host–guest interactions and reduce effective complexation [33,34,35,36].

The temperature also had a significant effect on the encapsulation efficiency. Under fixed conditions with the mass ratio of CCMLE to HP-*β*-CD set at 1:7, a stirring speed of 400 rpm and an inclusion time of 1 h, the inclusion efficiency increased from 20 °C to 30 °C, which was attributed to increased molecular motion and improved diffusion rates favouring host–guest interactions. However, further increases beyond 30 °C resulted in a decrease in the inclusion efficiency, possibly due to the thermal destabilization of the formed complexes or the partial degradation of thermolabile components [37,38,39].

The stirring speed had a marked effect on encapsulation. The optimum encapsulation efficiency was achieved at 300 rpm, suggesting that sufficient mixing at this speed promoted effective contact between HP-*β*-CD and the stilbenes. Higher stirring speeds may cause vortexing, foaming or even mechanical damage to the cyclodextrin cavity, which may displace already-complexed guest molecules [40,41,42].

The optimization of the reaction time showed that 2 h was sufficient to reach equilibrium and achieve the maximum encapsulation rate. Longer times did not provide a significant improvement, indicating that the complexation process was kinetically fast and reached saturation within this time.

Based on the above results, the optimal conditions for the preparation of the stilbene/HP-*β*-CD inclusion complex were determined as follows: a CCMLE to HP-*β*-CD mass ratio of 1:7, an inclusion temperature of 30 °C, a stirring speed of 300 rpm and a reaction time of 2 h.

#### 2.1.2. Optimization of Stilbene/HP-*β*-CD Inclusion Complex Preparation Using Response Surface Methodology (RSM)

Based on the results obtained from the single-factor experiments, three key factors, including the inclusion temperature (A), mass ratio of CCMLE to HP-*β*-CD (B) and stirring speed (C), were determined to be significantly influencing the inclusion efficiency. These factors were further evaluated using a three-level, three-factor Box–Behnken design (BBD) within the framework of the RSM to obtain the optimal preparation conditions. The coded and actual values for each variable are shown in Table 1, and the design matrix for the 17 experimental runs is outlined in Table 2. A quadratic regression model was developed to describe the relationship between the inclusion efficiency (Y) and the selected variables:

Y = 80.49 + 1.48A + 1.50B − 0.1238C + 0.2450AB + 0.4150AC − 0.8325BC − 3.05A^2^ − 1.83B^2^ − 1.87C^2^

To determine the inclusion efficiency, the prepared stilbene/HP-*β*-CD inclusion complex was dissolved in ethanol, assisted by sonication, and diluted to 10 mL. Prior to UPLC analysis, samples were filtered through a 0.22 μm membrane.

The results of the regression analysis are shown in Table S1. The model was statistically significant with a *p*-value < 0.01, indicating a high level of confidence. The coefficient of determination (R^2^) was 0.993, and the adjusted R^2^ (R_Adj_^2^) was 0.984, indicating a good fit between the experimental and predicted values. Furthermore, the precision of the model was 29.05, well above the threshold of 4, confirming the reliability and predictive ability of the model.

An analysis of variance (ANOVA) showed the relative influence of each factor on the inclusion efficiency. Among them, the mass ratio of CCMLE to HP-*β*-CD was the most significant factor, followed by the temperature and stirring speed. This was consistent with the trends observed in the single-factor experiments. The F-values for the interaction terms (AB, AC and BC) and quadratic terms (A^2^, B^2^, C^2^) further confirmed significant curvature in the response surface, suggesting the reliability of the model for optimizing the preparation of the HP-*β*-CD–stilbene inclusion complex.

**Figure 2:**
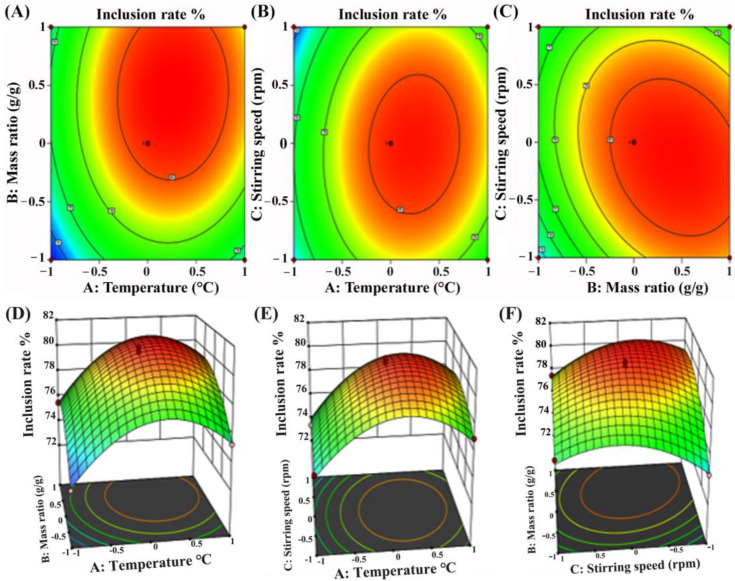
Contour and response surface plots illustrating interaction effects of process parameters on inclusion efficiency of stilbene/HP-*β*-CD inclusion complex: (**A**,**D**) interaction between inclusion temperature (**A**) and mass ratio of CCMLE to HP-*β*-CD (**B**) at fixed stirring speed; (**B**,**E**) interaction between inclusion temperature (**A**) and stirring speed (**C**) at fixed mass ratio; (**C**,**F**) interaction between mass ratio (**B**) and stirring speed (**C**) at fixed inclusion temperature.

The contour plots (Figure 2A–C) showed elliptical or saddle-shaped curves, indicating significant interactions between the variables, while the corresponding three-dimensional response surface plots (Figure 2D–F) exhibited curvature and the optimal region for achieving the maximum inclusion efficiency. It was indicated that the temperature played an important role in promoting the molecular interaction between stilbenes and HP-*β*-CD. At lower temperatures, reduced molecular mobility limited the penetration of stilbenes into the HP-*β*-CD cavity. In addition, the mass ratio had a more pronounced effect on the inclusion efficiency than the stirring speed, suggesting that adequate host availability is critical for effective inclusion. The optimal inclusion conditions predicted by the model were determined to be an inclusion temperature of 33 °C, a stirring speed of 272 rpm, a mass ratio of CCMLE to HP-*β*-CD of 1.0:8.2 (g/g) and a molar ratio of CCMLE to HP-*β*-CD of approximately 1:0.62. Under these optimized conditions, the inclusion efficiency was 82%.

### 2.2. Characterization of Stilbene/HP-β-CD Inclusion Complex

#### 2.2.1. Ultraviolet (UV) Spectroscopy Analysis

**Figure 3:**
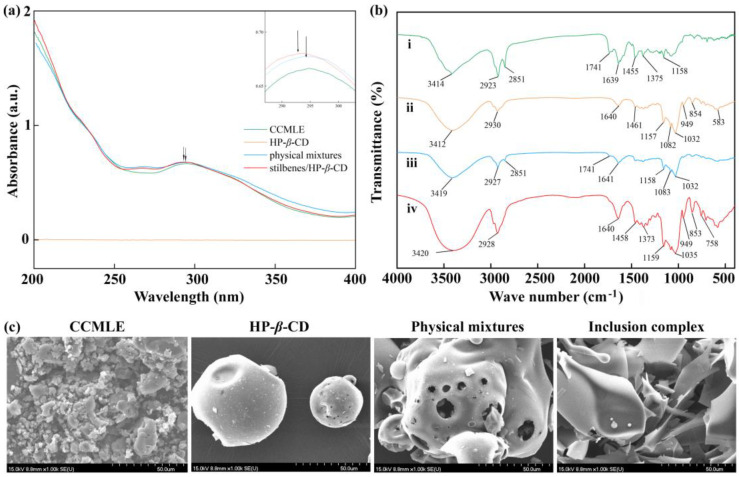
Characterization of stilbene/HP-*β*-CD inclusion complex. (**a**) UV absorption spectra of *Cajanus cajan* (L.) Millsp. leaf extract (CCMLE), HP-*β*-CD, physical mixtures of CCMLE and HP-*β*-CD, and stilbene/HP-*β*-CD inclusion complex; (**b**) FTIR spectra of (i) CCMLE, (ii) HP-*β*-CD, (iii) physical mixtures and (iv) stilbene/HP-*β*-CD inclusion complex; (**c**) scanning electron microscope (SEM) images of CCMLE, HP-*β*-CD, physical mixtures and stilbene/HP-*β*-CD inclusion complex.

As shown in Figure 3a, HP-*β*-CD had no significant absorption within the examined wavelength range of 200–400 nm. In contrast, both CCMLE and its physical mixture with HP-*β*-CD had characteristic absorption peaks at 295 nm, characteristic of stilbenes containing aromatic rings and conjugated double bonds. Upon inclusion in the HP-*β*-CD cavity, the characteristic absorption peaks did not change significantly, with only a slight blue shift in the maximum absorption wavelength. This shift can be attributed to hydrogen bonding and a change in polarity in the microenvironment surrounding the stilbenes. It was also suggested that the successful host–guest interactions did not result in chemical degradation.

#### 2.2.2. Fourier Transform Infrared (FTIR) Spectroscopy Analysis

The FTIR spectra of CCMLE, HP-*β*-CD, their physical mixture and the stilbene/HP-*β*-CD inclusion complex are shown in Figure 3b. CCMLE exhibited characteristic absorption bands at 3413 cm^−1^ (O-H stretching), 1741 cm^−1^ (C=O stretching from the presence of carboxylic acids) and 1455 cm^−1^ (C-H bending), while HP-*β*-CD showed its typical absorption bands at 3412 cm^−1^ (O-H stretching), 2930 cm^−1^ (C-H stretching of CH_2_) and 1032 cm^−1^ (C-O-C stretching), respectively, consistent with the literature [43]. The IR spectrum of the physical mixture basically retained the main absorption bands of both components, indicating no significant interaction [44].

In contrast, the FTIR spectrum of the inclusion complex closely resembled that of HP-*β*-CD, with a remarkable weaking of the absorption band at 1741 cm^−1^ and changes in the 1373~1458 cm^−1^ (C-H bending) region. These changes suggested that the inclusion complexes were formed through weak non-covalent interactions, which may have reduced the intensity of specific stilbene bands due to the stilbenes’ encapsulation within the HP-*β*-CD cavity [45].

#### 2.2.3. Scanning Electron Microscopy (SEM) Analysis

The SEM images (Figure 3c) revealed clear morphological differences between the samples. CCMLE appeared to contain large, irregular agglomerates, whereas HP-*β*-CD showed a relatively uniform spherical morphology. The physical mixture showed a simple combination of these two structures without morphological integration, suggesting a lack of interaction. In contrast, the stilbene/HP-*β*-CD inclusion complex displayed an irregular lamellar structure that differed significantly from the structure of the original components. This morphological transformation indicated that the molecular interactions between the host and guest led to structural reorganization during complex formation, suggesting the successful formation of a stilbene/HP-*β*-CD inclusion complex.

#### 2.2.4. Molecular Docking Analysis

**Figure 4:**
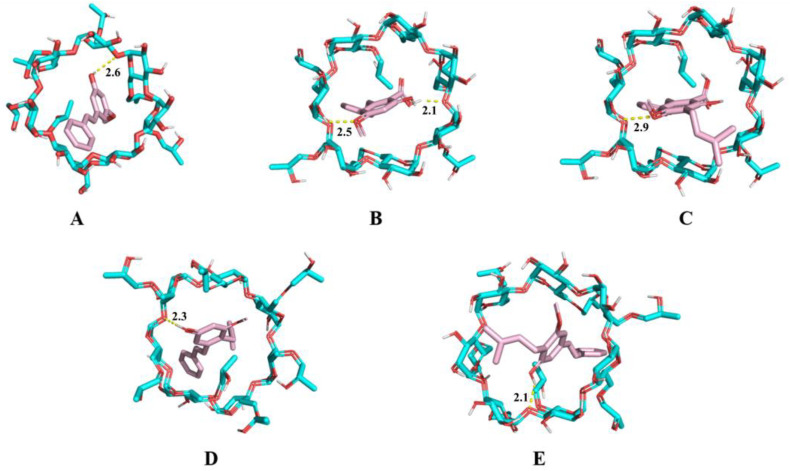
Molecular docking diagram of 2-hydroxypropyl-*β*-cyclodextrin (HP-*β*-CD) and pinosylvin monomethyl ether (PME) (**A**), 4-*O*-methylpinosylvic acid (MPA) (**B**), cajaninstilbene acid (CSA) (**C**), longistyle C (LLC) (**D**) and longistyle A (LLA) (**E**). Blue represents the structure of HP-*β*-CD, while pink represents stilbenes. The dotted line represents the hydrogen bond interaction formed by the two molecules.

To investigate the mechanisms of host–guest interaction between HP-*β*-CD and individual stilbene components, molecular docking was performed using AutoDock 4.2.6 software. Five major stilbenes identified in CCMLE, including CSA, LLC, MPA, LLA and PME, were docked into the HP-*β*-CD cavity (Figure 4). The calculated binding energies were −5.167 kcal/mol for PME, −5.384 kcal/mol for MPA, −5.165 kcal/mol for CSA, −5.059 kcal/mol for LLC and −5.545 kcal/mol for LLA. These negative binding energy values indicated that the process of the inclusion of stilbenes in HP-*β*-CD was a spontaneous, thermodynamically favourable process. The smaller the binding energy value, the stronger the binding force. Notably, all the binding energies were below −5 kcal/mol, indicating that these stilbenes can form relatively stable complexes with HP-*β*-CD. Based on these negative binding energies and the structural similarity of the studied stilbenes to resveratrol, which forms a 1:1 stoichiometric complex with sulfobutylether-*β*-cyclodextrin [29], we propose that the stilbene/HP-*β*-CD inclusion complex predominantly forms at a 1:1 stoichiometric molar ratio.

### 2.3. In Vitro Dissolution Study

**Figure 5:**
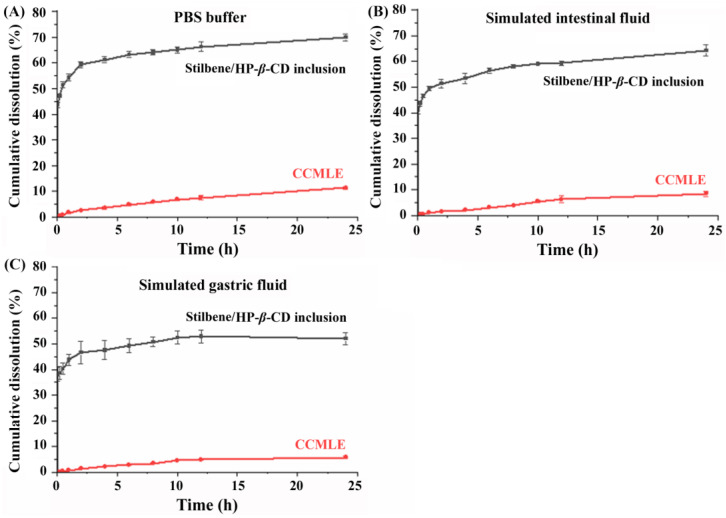
In vitro cumulative dissolution profiles of stilbenes from *Cajanus cajan* (L.) Millsp. leaf extract (CCMLE) and stilbene/HP-*β*-CD inclusion complexes in different media over 24 h (*n* = 3, mean ± SD): (**A**) pH 7.4 PBS buffer; (**B**) pH 6.8 simulated intestinal fluid (pH 6.8 SIF); (**C**) pH 1.2 simulated gastric fluid (pH 1.2 SGF).

To further investigate the dissolution behaviour of stilbenes before and after inclusion complex formation, in vitro release studies were performed using three different dissolution media: phosphate-buffered saline (PBS, pH 7.4), simulated gastric fluid (SGF) and simulated intestinal fluid (SIF). As shown in Figure 5, the solubility of the stilbenes was the highest in PBS, followed by SIF, with the lowest solubility observed in SGF. In neutral to mildly alkaline media (PBS, SIF), the partial deprotonation of the carboxyl group (CSA and MPA) enhanced the solubility. Additionally, the phenolic hydroxyl groups in these stilbenes, similar to those in resveratrol (pKa ~ 9.5–10), contributed to higher solubility in neutral conditions, though significant deprotonation requires highly alkaline environments (pH ~ 10).

The inclusion of the stilbenes in HP-*β*-CD significantly improved their dissolution rate compared to their presence in CCMLE alone. After 24 h, the cumulative release of stilbenes from the stilbene/HP-*β*-CD inclusion complex was 70% in PBS, 64% in SIF and 52% in SGF. In contrast, the dissolution rate of stilbenes from CCMLE was significantly slower (below 10%) in all the conditions tested. This improvement in both the rate and extent of dissolution suggested that the inclusion complex effectively enhanced the aqueous dispersion and release profile of the stilbenes, probably due to the increased wettability and improved dispersion afforded by the hydrophilic outer surface of HP-*β*-CD. In addition, the encapsulation may have maintained a dynamic equilibrium in which changes in the surrounding medium promoted the gradual release of free stilbene molecules [46].

To elucidate the dissolution mechanisms, the release data were fitted to four commonly applied kinetic models: the zero-order, first-order, Higuchi and Weibull models [47,48] (Table 3). The corresponding equations were as follows:

The zero-order equation:

(1) $$Q_{t}=Q_{0}+k_{0}\cdot t$$

The first-order equation:

(2) $$\ln\left(1-Q_{t}\right)={-k}_{1}\cdot t$$

The Higuchi equation:

(3) $$Q_{t}=k_{H}\cdot t^{\frac{1}{2}}$$

The Weibull equation:

(4) $$Q_{t}=1-e^{{-\left(\frac{t}{\tau }\right)}^{\beta }}$$

where *Q_t_* is the cumulative dissolution at time t, *Q*_0_ is the initial dissolution (usually 0), *k*_0_ is the zero-order dissolution rate constant, *k*_1_ is the first-order dissolution rate constant, *k_H_* is the Higuchi dissolution rate constant, *τ* is the scale parameter, representing the characteristic time taken to complete the dissolution process, and *β* is the shape parameter, describing the shape of the dissolution curve.

As summarized in Table 3, the dissolution profiles of stilbenes from the stilbene/HP-*β*-CD inclusion complex were best described by the Weibull equation in all three different media, with high regression coefficients—R^2^ values—of 0.995 in PBS, 0.989 in SIF and 0.985 in SGF. This consistency suggested that the inclusion complex had a controlled and predictable release mechanism, probably governed by diffusion and matrix relaxation processes.

However, the release kinetics of stilbenes from CCMLE varied depending on the dissolution medium. In the PBS buffer, the best fit was observed with the Higuchi model (R^2^ = 0.991), suggesting a diffusion-controlled release mechanism. In SIF, the data best fit the Weibull model (R^2^ = 0.990), suggesting a more complex release pattern, possibly influenced by solubilization and matrix erosion. In SGF, the release behaviour was best described by the first-order model (R^2^ = 0.990), reflecting a concentration-dependent release process. These results suggested that the release behaviour of stilbenes from CCMLE was more sensitive to environmental conditions such as the pH and enzymatic effects.

### 2.4. In Vivo Pharmacokinetic Studies in Rats

**Figure 6:**
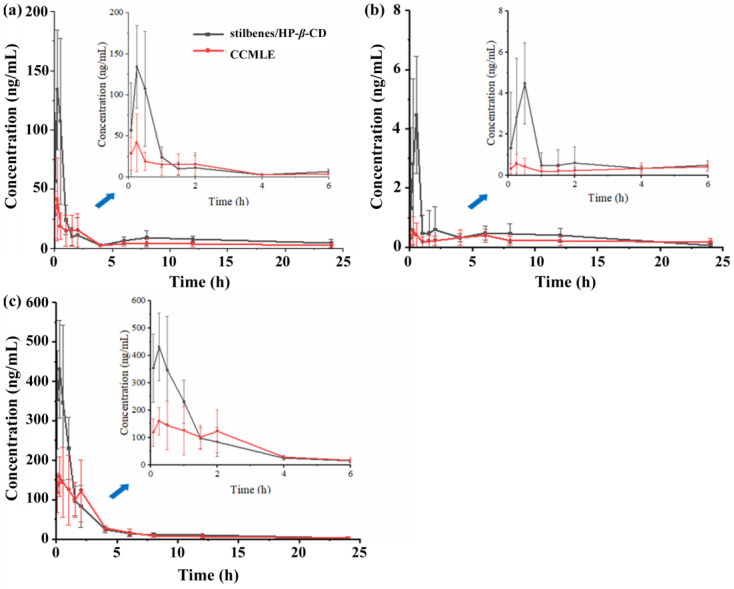
Mean plasma concentration–time profiles of cajaninstilbene acid (CSA) (**a**), longistyle C (LLC) (**b**) and 4-*O*-methylpinosylvic acid (MPA) (**c**) in rats following oral administration of *Cajanus cajan* (L.) Millsp. leaf extract (CCMLE) (red) and stilbene/HP-*β*-CD inclusion complex (black) over 24 h (*n* = 6, mean ± SD). Insets show detailed concentration changes within first 6 h.

The pharmacokinetic profiles of three representative stilbenes, CSA, LLC and MPA, were evaluated in rats following the oral administration of CCMLE or the corresponding stilbene/HP-*β*-CD inclusion complex. The plasma concentrations were determined using a validated UPLC-QqQ-MS/MS analytical method (Supplementary Figure S2 and Table S2). As shown in Figure 6, CSA and MPA reached peak plasma concentrations (*C_max_*) at 15 min, while the concentration of LLC peaked at 30 min post-administration. Notably, the maximum blood concentrations (*C_max_*) of all three stilbenes were significantly higher in rats given the stilbene/HP-*β*-CD inclusion complex than in those given CCMLE alone, indicating that complexation with HP-*β*-CD improved the bioavailability of stilbenes.

Pharmacokinetic parameters, including the *AUC*, *C_max_*, *t_max_* and *t*_1/2_, were calculated using DSA 2.0 software, and the results are shown in Table 4. Compared to CCMLE, the stilbene/HP-*β*-CD inclusion complex showed significantly increased *C_max_* and AUC_0→∞_ values for all three stilbenes, indicating improved oral bioavailability. Specifically, the *C_max_* increased for CSA from 48 ± 31 ng/mL to 140 ± 60 ng/mL, for LLC from 0.7 ± 0.5 ng/mL to 4.5 ± 2.0 ng/mL and for MPA from 230 ± 60 ng/mL to 440 ± 130 ng/mL. Similarly, the AUC_0→∞_ values were significantly higher in the inclusion complex group for CSA (470 ± 160 vs. 170 ± 66 ng-h/mL), LLC (12.0 ± 6.7 vs. 10.0 ± 9.3 ng-h/mL) and MPA (803 ± 140 vs. 580 ± 170 ng-h/mL). The calculated relative bioavailability of CSA, LLC and MPA from the inclusion complex, using CCMLE as a reference, was 198%, 177% and 131%, respectively.

This improvement in bioavailability can be attributed to the enhanced aqueous solubility conferred by HP-*β*-CD encapsulation, which is likely to have facilitated greater absorption in the gastrointestinal tract. Cyclodextrin inclusion complexes maintain drugs in a dissolved state, thus promoting higher permeability across the intestinal epithelium [49,50].

Interestingly, differences in the elimination half-life (*t*_1/2_) were also observed. For CSA and MPA, the *t*_1/2_ was slightly prolonged in the inclusion group, suggesting a slower elimination rate and prolonged systemic exposure. However, for LLC, the *t*_1/2_ was shortened (6 h vs. 14 h). As shown in Figure 6b, the concentration–time curve of LLC in the CCMLE group exhibited a bimodal pattern, with an initial peak at 30 min followed by a decline and subsequent increase. This may have been due to enterohepatic recirculation or delayed redistribution from tissue compartments. The absence of this secondary peak in the inclusion complex group, together with a significantly elevated initial *C_max_*, suggested that encapsulation may have accelerated the initial absorption phase, reducing the extent of subsequent redistribution and thereby shortening the apparent *t*_1/2_.

### 2.5. Tissue Distribution

**Figure 7:**
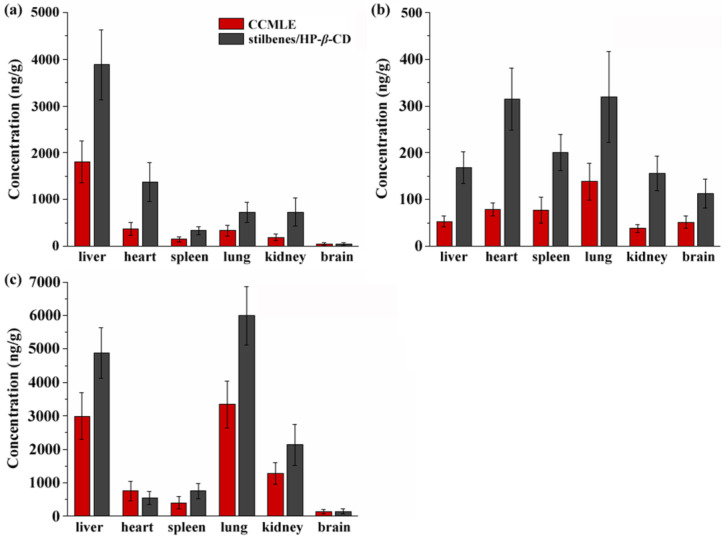
Tissue distribution of cajaninstilbene acid (CSA) (**a**), longistyle C (LLC) (**b**) and 4-*O*-methylpinosylvic acid (MPA) (**c**) in rats after oral administration of *Cajanus cajan* (L.) Millsp. leaf extract (CCMLE) and stilbene/HP-*β*-CD inclusion complex. Concentrations in heart, liver, spleen, lungs and kidneys were determined 1 h after administration (*n* = 6, mean ± SD).

The tissue distribution profiles of the three stilbenes following the oral administration of either CCMLE or the stilbene/HP-*β*-CD inclusion complex were assessed in different rat tissues. As shown in Figure 7, all three of these compounds were detectable in the heart, liver, spleen, lungs and kidneys and brains, indicating a broad systemic distribution.

Although the overall tissue distribution pattern was similar between the CCMLE and inclusion complex groups, distinct tissue-specific accumulation characteristics were observed for the individual stilbenes. CSA was predominately concentrated in the liver, suggesting that liver tissue may be a major site of its metabolism or action. In contrast, LLC showed higher concentrations in the heart and lungs, possibly reflecting differences in its tissue affinity or permeability. MPA exhibited relatively higher levels in the liver, lungs and kidneys, indicating a wider distribution and possibly greater systemic exposure.

Importantly, for all three stilbenes, the tissue concentrations were consistently higher in the inclusion complex group compared to the CCMLE group. This observation suggested that the HP-*β*-CD encapsulation not only improved gastrointestinal absorption but also facilitated increased tissue penetration and retention. The improved distribution may have been due to the increased solubility and stability of the stilbenes in their inclusion complex form, which may have reduced metabolic degradation and promote prolonged circulation and tissue uptake.

Despite the promising results, this study has limitations. Our focus on using HP-*β*-CD for encapsulation meant that we did not examine other cyclodextrin types, such as α- or γ-cyclodextrin, which could affect the stilbene solubility and in vivo absorption differently. Furthermore, the in vivo mechanisms of action of stilbene/HP-*β*-CD complexes require further exploration using disease-specific models. However, the established method offers a pathway for future studies to investigate these aspects and optimize stilbene delivery.

## 3. Materials and Methods

### 3.1. Chemicals and Materials

Longistylin C (≥98%) was obtained from Tianzhi Biotechnology (Wuhan, China). HP-*β*-CD (≥98%) was purchased from Taitan Science and Technology (Shanghai, China). HPLC-grade acetonitrile was supplied by Merck (Darmstadt, Germany). Formic acid, pepsin and trypsin were purchased from Aladdin Biochemical Technology Co., Ltd (Shanghai, China). Methanol was purchased from the Tengyu New Material Technology company (Zhejiang, China). Freeze-dried phosphate-buffered saline (PBS) powder was purchased from Baisha biotechnology (Hefei, China). Ultrapure water (18.2 MΩ) was prepared using a Milli-Q IQ 7000 water purification system (Darmstadt, Germany). CCMLs were kindly provided by the Guangxi Wushengyuan Agricultural Development Co., LTD. (Nanning, China).

### 3.2. Preparation of Stilbene-Enriched Cajanus cajan (L.) Millsp. Leaf Extract (CCMLE)

The dried CCML samples were ground into a fine powder and sieved through a 40-mesh sifter. Then 5 g of the powdered sample was mixed with 200 mL of pure water and subjected to reflux extraction for 1 h. After cooling to room temperature, the mixture was filtered, and the filter residue was dried in an oven at 40 °C. The dried residue was then weighed and extracted with methanol (1:40, *w*/*v*) under ultrasound at 30 °C for 20 min. The resulting extract was filtered, concentrated by evaporation and lyophilized to obtain stilbene-enriched CCMLE.

### 3.3. Preparation and Optimization of Stilbene/HP-β-CD Inclusion Complex

#### 3.3.1. The Preparation of the Stilbene/HP-β-CD Inclusion Complex

Firstly, 50 mg of CCMLE was dissolved in ethanol as the organic phase, and 450 mg of HP-*β*-CD was dispersed in ultrapure water as the aqueous phase. The organic phase was added dropwise to the aqueous phase under magnetic stirring at 50 °C and 400 rpm for 1 h. After cooling to room temperature, the ethanol was removed from the mixture. Subsequently, centrifugation was performed at 10,000 rpm for 10 min at 4 °C to remove any residual free stibene compounds, and the supernatant was collected and lyophilized to obtain the stilbene/HP-*β*-CD inclusion complex powder.

#### 3.3.2. Preparation and Optimization of Stilbene/HP-β-CD Inclusion Complex Using Single-Factor Method

Single-factor experiments were conducted to investigate the influence of different factors on the inclusion efficiency of stilbenes. The effects of the mass ratio of CCMLE to HP-*β*-CD (1:1, 1:3, 1:5, 1:7 and 1:9), reaction temperature (20, 30, 40, 50 and 60 °C), magnetic stirring speed (200, 300, 400, 500 and 600 rpm) and reaction time (1, 2, 3, 4 and 5 h) were evaluated. Each factor was examined independently while keeping the other conditions constant to determine its effect on the stilbene content within the inclusion complex.

#### 3.3.3. Optimization of Stilbene/HP-β-CD Inclusion Complex Using Response Surface Methodology (RSM)

Based on the results of the single-factor experiment, the three factors that had the greatest influence on the inclusion efficiency were selected for investigation in a three-factor, three-level response surface experiment. The response surface optimization data were processed with design expert 13 to optimize the preparation conditions.

### 3.4. Ultra-Performance Liquid Chromatography (UPLC) Analysis Conditions

The quantification of the stilbenes was conducted by using a Waters H-Class UPLC system (Waters, Milford, MA, USA). Separation was performed on a Waters BEH Shield RP 18 column (Waters, Milford, MA, USA, 100 × 2.1 mm, 1.7 μm) at 35 °C. The mobile phase consisted of 0.1% formic acid in water (solvent A) and acetonitrile (solvent B), with a flow rate of 0.2 mL/min. A gradient elution programme was used as follows: 0–2 min, 95–90% A; 2–3 min, 90–85% A; 3–12 min, 85% A; 12–18 min, 85–60% A; 18–24 min, 60% A; 24–27 min, 60–40% A; 27–41 min, 40% A; 41–42 min, 40–95% A; and 42–45 min, 95% A. The detection wavelength was set at 305 nm. The injection volume was 2 µL.

### 3.5. Preparation of Longistyle C (LLC) Standard Solution for Stilbene Quantification

An accurately weighed amount of 9.14 mg LLC was dissolved in a 25 mL volumetric flask and diluted to the mark with ethanol to prepare a standard stock solution (365.60 μg/mL). The stock solution was then diluted 50-, 20-, 10-, 8- and 5-fold with ethanol to prepare a series of working standard solutions for calibration curve construction. All the solutions were stored at 4 °C until UPLC analysis. The content of the stilbenes in the samples was quantified using the LLC reference standard calibration curve.

### 3.6. Determination of Inclusion Efficiency

In this study, the content of five kinds of stilbenes, including CSA, LLC, MPA, longistyline A (LLA) and pinosylvin monomethyl ether (PME), was summed to represent the total content of stilbenes in both the stilbene/HP-*β*-CD inclusion complex and CCMLE. The prepared stilbene/HP-*β*-CD inclusion complex was dissolved in ethanol and ultrasonicated to ensure complete dissolution. The resulting solution was then adjusted to a final volume of 10 mL with ethanol. Prior to ultra-high-performance liquid chromatography (UPLC) analysis, the solution was filtered through a 0.22 μm microporous membrane. The formula for calculating the inclusion efficiency was as follows:

(5) $$Inclusion\;rate\;(\%)=\frac{m_{A\;}(mg)}{m_{B}\;(mg)}\times 100\%$$

where *m_A_* represents the mass of stilbenes contained in the inclusion complex and *m_B_* represents the mass of stilbenes in the initially added CCMLE. The concentration of the stilbenes in the stilbene/HP-*β*-CD inclusion complex was calculated by drug loading using the following formula:

(6) $$Drug\;loading(\%)=\frac{m_{i}(mg)}{m_{stilbenes/HP-\beta -CD}(mg)}\times 100\%$$

where *m*_i_ represents the amount of stilbenes in the stilbene/HP-*β*-CD inclusion complex quantified by UPLC and *m*_stilbenes/HP-_*_β_*_-CD_ represents the mass of the inclusion complex.

### 3.7. Water Solubility of Stilbene/HP-β-CD Inclusion Complex

An excess amount of CCMLE (200 mg) or the stilbene/HP-*β*-CD inclusion complex (500 mg) was added to water (10 mL). The suspension was then stirred (200 rpm) at 25 °C for 48 h. At 2, 6, 10, 24 and 48 h, 1 mL of the solution was taken and then centrifuged at 3200× *g* for 10 min, and the supernatant was analyzed by UPLC.

### 3.8. Characterization Using Ultraviolet (UV) Spectroscopy

Appropriate amounts of CCMLE, HP-*β*-CD, the CCMLE + HP-*β*-CD physical mixture and the stilbene/HP-*β*-CD inclusion complex were taken separately and dissolved in water to obtain the sample solutions. The UV absorption spectra of these samples were recorded using a Shimadzu UV-2250 spectrophotometer (Shimadzu, Kobe, Japan) with a wavelength range of 200–400 nm.

### 3.9. Characterization via Fourier Transform Infrared (FTIR) Spectroscopy

CCMLE, HP-*β*-CD, the CCMLE + HP-*β*-CD physical mixture and the stilbene/HP-*β*-CD inclusion complex were individually mixed with potassium bromide (KBr) at a ratio of 1:30 (*w*/*w*). Each mixture was finely ground and pressed into transparent pellets. FTIR spectra were acquired using a Thermo Scientific Nicolet iS50 FTIR spectrometer (Thermo Fisher Scientific, Waltham, MA, USA) within the range of 4000–400 cm^−1^ with a resolution of 2 cm^−1^.

### 3.10. Morphological Characterization by Scanning Electron Microscopy (SEM)

The surface morphology of CCMLE, HP-*β*-CD, the CCMLE + HP-*β*-CD physical mixture and the stilbene/HP-*β*-CD inclusion complex was characterized using SEM (S-4700, Hitachi, Tokyo, Japan). The solid samples were evenly dispersed onto conductive tape, followed by platinum coating (200 s) using a sputter coater to enhance the conductivity. Imaging was performed at an acceleration voltage of 15.0 kV.

### 3.11. Molecular Docking Analysis

The binding interactions between HP-*β*-CD and five stilbenes were investigated using AutoDock 4.2.6 software. The three-dimensional structures of both the receptor HP-*β*-CD and the ligand stilbenes were obtained from the ChemSpider database using their respective CAS numbers. Prior to docking, the structures were prepared by removing water molecules, adding hydrogen atoms and optimizing the charge distribution to ensure accurate simulations.

### 3.12. Preparation of Simulated Fluids and In Vitro Dissolution Study

Simulated gastric fluid (SGF, pH 1.2) was prepared by diluting 16.4 mL of 0.1 M HCl in 1000 mL of distilled water and adjusting the pH to 1.2. Then 10 g of pepsin was added to the SGF.

Simulated intestinal fluid (SIF, pH 6.8) was prepared by first dissolving 6.8 g of KH_2_PO_4_ in 500 mL of distilled water and adjusting the pH to 6.8 using 0.1 M NaOH. Then 10 g of trypsin was dissolved in an appropriate amount of water and added to the KH_2_PO_4_ buffer. The final volume was adjusted to 1000 mL with distilled water.

In addition, phosphate-buffered saline (PBS, pH 7.4) was prepared by dissolving 20 g of PBS powder in 2 L of distilled water.

The in vitro dissolution of CCMLE and the stilbene/HP-*β*-CD inclusion complex (each containing 46.40 mg CCMLE) was evaluated using the basket method. Samples were placed in 900 mL of dissolution media (SGF, SIF and PBS) at 37 °C under constant agitation (100 rpm). Aliquots containing 2 mL of the samples were withdrawn at predetermined intervals at 5, 15, 30 and 45 min, followed by 1, 2, 4, 6, 8, 10, 12 and 24 h, with immediate replenishment with an equal volume of fresh media to maintain sink conditions. The withdrawn samples were processed and analyzed by UPLC (as described in Section 3.5), and the cumulative dissolution of the stilbenes was calculated using the following formula:

(7) $$\mathrm{Cumulative}\;\mathrm{dissolution}\;\mathrm{rate}\;(\%)=\frac{M_{t}+{2\;\times \;C}_{t-1}}{M_{0}}\;\times \;100$$

where *M_t_* is the mass of the cumulative dissolved stilbenes at the corresponding sampling point, *C_t_*_−1_ is the concentration of the stilbenes at the previous sampling point and *M*_0_ is the mass of the initially added stilbenes.

### 3.13. In Vivo Pharmacokinetic Studies in Rats

#### 3.13.1. Animals and Experimental Design

Healthy male SD rats weighing 200 ± 20 g were housed in a specific pathogen-free (SPF) facility under controlled conditions (24 ± 1 °C, 50 ± 10% relative humidity) with a 12 h light/dark cycle. The animals were free to access purified water and a standard laboratory diet for a 1-week acclimatization period. After acclimatization, the rats were randomly divided into three groups (*n* = 6 per group): the control group (con), CCMLE group and stilbene/HP-*β*-CD inclusion complex group. The CCMLE group received an oral dose of 232 mg/kg CCMLE, while the stilbene/HP-*β*-CD inclusion complex group received 2.1 g/kg of the inclusion complex by oral gavage, with both containing the same amount of stilbenes. All animal experiments were performed in accordance with the National Institutes of Health Guide for the Care and Use of Laboratory Animals (NIH Publication No. 85-23, revised in 1996). The study protocol was approved by the Animal Care and Use Committee of Zhejiang University of Technology (approval number MGS20231227194).

#### 3.13.2. Blood Sample Collection and Processing

Blood samples (approximately 300 μL) were collected via the retro-orbital venous plexus before dosing and at the following post-dose times of 5 min, 15 min, 30 min, 1 h, 1.5 h, 2 h, 4 h, 6 h, 8 h, 12 h and 24 h. Each sample was placed in an EDTA anticoagulation tube to prevent coagulation. The tubes were then centrifuged at 3500 rpm for 10 min. The upper layer of supernatant (plasma) was carefully collected and stored at −80 °C until further analysis.

#### 3.13.3. Plasma Sample Preparation

The plasma samples stored at −80 °C were thawed at room temperature prior to processing. For each analysis, 100 μL of the plasma sample was accurately transferred into a centrifuge tube, followed by the addition of 40 μL of a chloramphenicol (CHL) solution (1 mg/mL) used as an internal standard (IS) and 260 μL methanol. The mixture was vortexed for 3 min to ensure thorough homogenisation and then centrifuged at 13,000 rpm for 10 min at 4 °C to precipitate proteins and other insoluble components. For analysis, 10 μL of the supernatant was injected into a ultra-high-performance liquid chromatography–triple-quadrupole tandem mass spectrometry (UPLC-QqQ-MS/MS) system.

#### 3.13.4. Bioavailability Determination of Stilbenes

The bioavailabilities of three stilbenes (LLC, CSA and MPA) in CCMLE and the stilbene/HP-*β*-CD inclusion complex were analyzed using a Waters Xevo TQ-S micro-mass spectrometry system (Waters, Milford, MA, USA) coupled to a Waters H-Class UPLC system. Chromatographic separation was performed on a Waters H-Class UPLC system equipped with a Welch Ultimate^®^ UHPLC LP-C18 column (Waters, Milford, MA, USA, 2.1 × 50 mm, 1.8 μm) at 30 °C. The injection volume was 10 μL. The mobile phase consisted of ultrapure water (A) and acetonitrile (B) with a flow rate of 0.3 mL/min and an elution gradient programme as follows: 0–0.2 min, 90% A; 0.2–1.5 min, 90–5% A; 1.5–3.5 min, 5% A; and 3.5–5 min, 5–90% A.

Mass spectrometric analysis was performed on a Waters Xevo TQ-S micro system using electrospray ionization in the negative mode (ESI^−^). The capillary voltage was 2.71 kV, and the ion source temperature was 150 °C. Nitrogen was used as the desolventizing gas and the cone pore gas, and argon was used as the collision gas. Data acquisition was performed in the multiple-reaction-monitoring (MRM) mode for all analytes. The optimized MRM conditions are listed in Table 5.

The relative bioavailability [51,52] of CSA, LLC and MPA in the stilbene/HP-*β*-CD inclusion complex was calculated using Equation (8), with CCMLE used as a reference:

(8) $$F_{rel}=\frac{{AUC}_{stilbenes/HP-\beta -CD}}{{AUC}_{CCMLE}}\cdot \frac{D_{CCMLE}}{D_{stilbenes/HP-\beta -CD}}\cdot 100\%$$

where *AUC*_stilbenes/HP-*β*-CD_ and *AUC*_CCMLE_ represent the areas under the blood concentration–time curve, and *D*_stilbenes/HP-*β*-CD_ and *D*_CCMLE_ represent the orally administered dose (mg/kg).

#### 3.13.5. Methodology Validation for Ultra-High-Performance Liquid Chromatography–Triple-Quadrupole Tandem Mass Spectrometry (UPLC-QqQ-MS/MS)

(1) Specificity

Blank plasma, blank plasma spiked with reference standards and CHL, and plasma samples collected 30 min after the oral administration of the stilbene/HP-*β*-CD inclusion complex spiked with CHL were analyzed by UPLC-QqQ-MS/MS. The specificity was assessed by comparing the chromatograms of the above three groups of samples to confirm the absence of endogenous interference in the analyte retention times.

(2) Linearity and range

A mixed standard stock solution was prepared in methanol with concentrations of 52.65 (LLC), 57.04 (CSA), 34.38 (MPA) and 52.10 (CHL) μg/mL. Serial dilutions were performed to achieve working solutions with concentrations of LLC of 0.17–105.30 ng/mL, CSA of 0.18–570.38 ng/mL and MPA of 0.11–687.38 ng/mL. The prepared working solutions were injected into a UPLC-QqQ-MS/MS system for analysis. Calibration curves were plotted with the concentration of the analyte (X) against the peak area ratio of the analyte to IS CHL (Y), and linear regression was performed to determine the equation and correlation coefficient (r).

(3) The lowest limits of quantification (LLOQs)

The LLOQ was determined at the concentration with a signal-to-noise ratio of 10, which was also the lowest concentration on the calibration curve.

(4) Precision and accuracy

The precision and accuracy of the method were evaluated by analyzing quality control (QC) samples at three concentration levels (high, medium and low) in six replicates per level per day for three consecutive days. The intra-day (within-day) and inter-day (between-day) precision were expressed as the relative standard deviation (RSD). The accuracy was expressed as the relative error (RE) between the measured and nominal concentrations. The method was considered acceptable if the RSD for precision and the RE for accuracy were within ±15% at all concentration levels.

(5) Recovery and matrix effects

The extraction recovery and matrix effect were assessed using QC samples at low, medium and high concentration levels. To assess the recovery, the peak areas of the analytes obtained from the QC samples (group Q) were compared with those obtained from the samples spiked post-extraction (group P), which comprised blank plasma that had been extracted and then spiked with the analytes at the appropriate concentrations. The recovery (%) was calculated as the ratio of the peak area of group Q to that of group P multiplied by 100.

To assess the matrix effect, the peak areas of the samples spiked after extraction (group P) were compared with those of pure standard solutions prepared in methanol at the same concentrations (group M). The matrix effect (%) was calculated as the ratio of the peak area of group P to that of group M multiplied by 100. All the experiments were performed in six replicates (*n* = 6) for each concentration level.

(6) Stability

The stability of the analytes in plasma was evaluated under different conditions at low, medium and high QC concentrations. The short-term stability was assessed by storing QC samples at room temperature (25 °C) for 24 h prior to analysis. The long-term stability was assessed by storing QC samples at −20 °C for 20 days. The freeze–thaw stability was determined after three freeze–thaw cycles, with samples frozen at −20 °C and thawed at room temperature for each cycle. Following the specified storage or handling conditions, the samples were analyzed and the measured concentrations compared with those of freshly prepared QC samples. The stability was considered acceptable if the relative error (RE) was within ±15% and the relative standard deviation (RSD) did not exceed 15%.

#### 3.13.6. Pharmacokinetic Analysis

DAS 2.0 software was used to calculate the pharmacokinetic parameters, including the area under the plasma concentration–time curve (*AUC*), maximum plasma concentration (*C_max_*), time to reach *C_max_* (*t_max_*) and elimination half-life (*t*_1/2_), using a non-compartmental model. All the data were statistically analyzed and plotted using Origin Pro 2024. Uncertainties were reported to two significant figures.

### 3.14. Tissue Distribution Study

The tissue distribution study followed the same experimental grouping and dosing protocols described in Section 3.12. Thirty minutes after dosing, the rats were euthanized and their tissues (including their heart, liver, spleen, lungs, kidneys and brain) were immediately collected. Each tissue was rinsed with a 0.9% NaCl solution, gently dried with filter paper, weighed and stored at −80 °C until analysis.

For sample preparation, 0.2 g of each tissue was homogenized in 0.5 mL of a 0.9% NaCl solution using a high-throughput tissue grinder (Jingxin, Shanghai, China). Next, 300 μL of the tissue homogenate was mixed with 50 μL of a CHL solution and 850 μL of methanol, vortexed for 3 min and centrifuged at 13,000 rpm for 10 min at 4 °C. Then 10 μL of the supernatant was injected into the UPLC-QqQ-MS/MS system for analysis.

## 4. Conclusions

In this study, a stilbene/HP-*β*-CD inclusion complex was successfully developed using HP-*β*-CD for the encapsulation of stilbenes in *Cajanus cajan* (L.) Millsp. leaf extracts (CCMLEs). The inclusion complex demonstrated its ability to significantly improve the solubility, dissolution behaviour, bioavailability and tissue distribution of three representative stilbenes (CSA, LLC and MPA). The dissolution kinetics showed that HP-*β*-CD encapsulation resulted in a more consistent and medium-independent release mechanism, as reflected by the good fit with the Weibull model across different simulated gastrointestinal environments. Pharmacokinetic analysis showed that the inclusion complex significantly increased both the *C_max_* and AUC_0→∞_ values of the stilbenes, with relative bioavailability increases of up to 198%. Tissue distribution studies further confirmed that the inclusion complex promoted the broader and more efficient absorption of the compounds into major organs, particularly the liver, lungs and kidneys. These improvements are likely to have been due to the increased solubility and reduced metabolic degradation afforded by HP-*β*-CD encapsulation. The HP-*β*-CD inclusion complex improved both the bioavailability and tissue distribution of stilbenes, which may have contributed to their enhanced pharmacological efficacy in vivo. This work provides a promising strategy for enhancing the oral delivery and systemic exposure of poorly soluble active compounds from traditional Chinese medicines by forming inclusion complexes.

## Figures and Tables

**Table 1:** Coded and actual levels of independent variables applied using response surface methodology for optimization of stilbene/HP-*β*-CD inclusion complex preparation.

Factor	Level		
	−1	0	1
A: Temperature (°C)	20	30	40
B: Mass ratio (g/g)	1:5	1:7	1:9
C: Stirring speed (rpm)	200	300	400

**Table 2:** Experimental design and inclusion efficiency results obtained using response surface methodology.

Run No.	Temperature (°C)	Mass Ratio (g/g)	Stirring Speed (rpm)	Inclusion Efficiency (%)
1	0	1	−1	79
2	0	−1	−1	74
3	0	0	0	81
4	0	0	0	80
5	0	0	0	80
6	1	−1	0	75
7	1	0	1	77
8	−1	0	−1	75
9	0	0	0	81
10	0	−1	1	76
11	−1	−1	0	73
12	1	1	0	79
13	−1	0	1	73
14	−1	1	0	75
15	1	0	−1	77
16	0	0	0	80
17	0	1	1	77

**Table 3:** Regression coefficients (R^2^) of different kinetic models fitted to dissolution data of stilbenes from *Cajanus cajan* (L.) Millsp. leaf extract (CCMLE) and stilbene/HP-*β*-CD inclusion complex in different media.

Model	Regression Coefficient (R^2^)					
	pH 7.4 PBS Buffer		Simulated Intestinal Fluid		Simulated Gastric Fluid	
	Inclusion Complex	CCMLE	Inclusion Complex	CCMLE	Inclusion Complex	CCMLE
Zero-order equation	0.249	0.937	0.267	0.934	0.183	0.844
First-order equation	0.881	0.989	0.875	0.986	0.891	0.990
Higuchi equation	0.482	0.991	0.486	0.961	0.406	0.963
Weibull equation	0.995	0.984	0.989	0.990	0.985	0989

**Table 4:** Pharmacokinetic parameters of cajaninstilbene acid (CSA), longistyle C (LLC) and 4-*O*-methylpinosylvic acid (MPA) in rats after oral administration of *Cajanus cajan* (L.) Millsp. leaf extract (CCMLE) and stilbene/HP-*β*-CD inclusion complex.

Parameter	CSA		LLC		MPA	
	CCMLE	Inclusion Complex	CCMLE	Inclusion Complex	CCMLE	Inclusion Complex
*t*_1/2_/h	11.0 ± 5.6	13.0 ± 9.9	14.0 ± 8.7	6.0 + 2.6	5.5 ± 1.7	9.0 ± 4.1
*t_max_*/h	0.3 ± 0.1	0.3 ± 0.1	0.3 ± 0.1	0.5 ± 0.0	0.4 ± 0.3	0.3 ± 0.1
*C_max_*/(ng/mL)	48 + 31	140 ± 60	0.7 ± 0.5	4.5 ± 2.0	230 ± 60	440 ± 130
AUC_0→t_/(ng·h/mL)	120 ± 41	240 ± 82	5.8 ± 2.9	10.0 ± 6.7	560 ± 160	740 ± 100
AUC_0→∞_/(ng·h/mL)	170 ± 66	470 ± 160	10.0 ± 9.3	12.0 ± 6.7	580 ± 170	803 ± 140

**Table 5:** Mass spectrometric parameters in multiple-reaction-monitoring mode.

Compound	Formula	Parent Ion	Daughter Ion	Cone (V)	Collision Energy (V)
CHL	C_11_H_12_Cl_2_N_2_O_5_	321.04	151.95	25	18
LLC	C_20_H_22_O_2_	293.13	235.08	40	23
CSA	C_21_H_22_O_4_	337.14	223.14	30	28
MPA	C_16_H_14_O_4_	269.08	210.04	40	22

## Data Availability

The original contributions presented in this study are included in the article. Further inquiries can be directed to the corresponding authors.

## Supplementary Materials

The following supporting information can be downloaded at: https://www.mdpi.com/article/10.3390/molecules30122526/s1, Figure S1. The effects of mass ratio (A), temperature (B), stirring speed (C) and reaction time (D) on the inclusion rate of stilbenes. Figure S2. MRM chromatograms: (A). blank plasma; (B). blank plasma with addition of control standards and chloramphenicol (CHL); (C). plasma samples following oral administration of stilbene/HP-*β*-CD inclusion complex after 30 min with addition of CHL. Table S1. Analysis of variance (ANOVA) for the response surface model to evaluate the effects of temperature, mass ratio and stirring speed on the inclusion efficiency of stilbenes. Table S2. UPLC-QqQ-MS/MS analytical method validation: The regression equation, precision, accuracy, extraction recoveries, matrix effects and stability of cajaninstilbene acid (CSA), longistyle C (LLC) and 4-*O*-methylpinosylvic acid (MPA) in rat plasma.
