# Supplementary material for: Improved Bioavailability of Stilbenes from Cajanus cajan (L.) Millsp. Leaves Achieved by Hydroxypropyl-β-Cyclodextrin Inclusion: Preparation, Characterization and Pharmacokinetic Assessment
Source: Molecules. 2025 Jun 10;30(12):2526. doi: 10.3390/molecules30122526 (PMC12195777; doi:10.3390/molecules30122526)
Supplement: Supplementary file 1 [file molecules-30-02526-s001.zip › molecules-3661624-supplementary.pdf]

Supporting information

Improved Bioavailability of Stilbenes from *Cajanus cajan* (L.) Millsp. Leaves Achieved by Hydroxypropyl-*β*-Cyclodextrin Inclusion: Preparation, Characterization and Pharmacokinetic Assessment

Yingya Qiu [1], Jiangxuan Lai [1], Yuhan Zhang [1], Sheng Fang [2], Zili Guo [3,*] and Xianrui Liang [1,*]

[1] Key Laboratory for Green Pharmaceutical Technologies and Related Equipment of Ministry of Education, College of Pharmaceutical Sciences, Zhejiang University of Technology, Hangzhou 310014, China; qiuyingya0326@163.com (Y.Q.); laijianngxuan@163.com (J.L.); zhangyuhan2001619@163.com (Y.Z.)
[2] School of Food Science and Biotechnology, Zhejiang Gongshang University, Hangzhou 310018, China; fangsheng@zjgsu.edu.cn
[3] Key Laboratory of Pollution Exposure and Health Intervention of Zhejiang Province, Zhejiang Collaborative Innovation Center for Full-Process Monitoring and Green Governance of Emerging Contaminants, Interdisciplinary Research Academy, Zhejiang Shuren University, Hangzhou 310015, China
* Correspondence: guozili@zjsru.edu.cn (Z.G.); liangxrvicky@zjut.edu.cn (X.L.)

Figure S1. The effects of mass ratio (A), temperature (B), stirring speed (C) and reaction time (D) on the inclusion rate of stilbenes

Figure S2. MRM chromatograms: A. blank plasma; B. blank plasma with addition of control standards and chloramphenicol (CHL); C. plasma samples following oral administration of stilbenes/HP-*β*-CD inclusion complex after 30 min with addition of CHL

Table S1. Analysis of variance (ANOVA) for the response surface model to evaluate the effects of temperature, mass ratio and stirring speed on the inclusion efficiency of stilbenes

Table S2. UPLC-QqQ-MS/MS analytical method validation: The regression equation, precision, accuracy, extraction recoveries, matrix effects and stability of cajaninstilbene acid (CSA), longistyle C (LLC) and 4-*O*-methylpinosylvic acid (MPA) in rat plasma

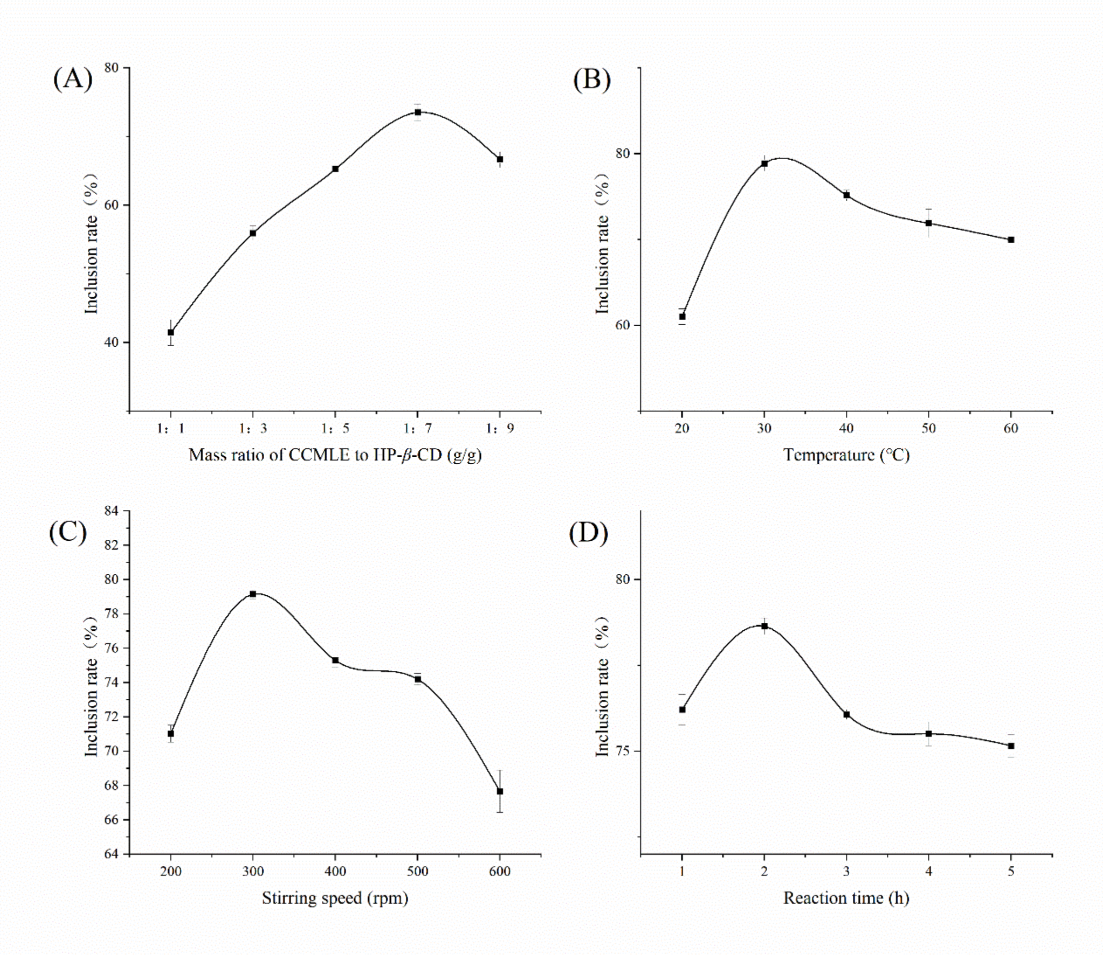

Figure S1. The effects of mass ratio (A), temperature (B), stirring speed (C) and reaction time (D) on the inclusion rate of stilbenes

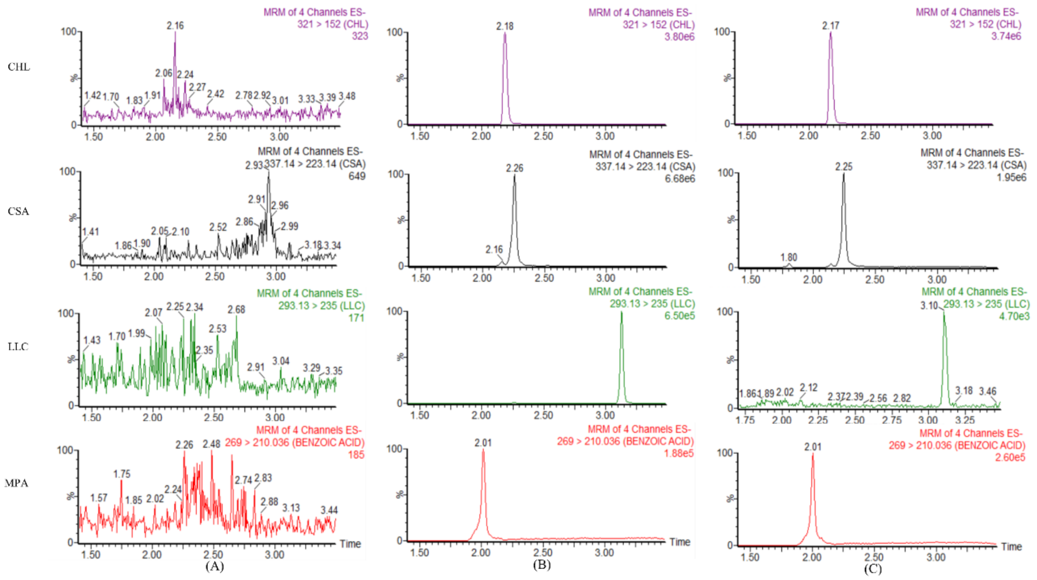

Figure S2. MRM chromatograms: A. blank plasma; B. blank plasma with addition of control standards and chloramphenicol (CHL); C. plasma samples following oral administration of stilbenes/HP-β-CD inclusion complex after 30 min with addition of CHL

Table S1. Analysis of variance (ANOVA) for the response surface model to evaluate the effects of temperature, mass ratio and stirring speed on the inclusion efficiency of stilbenes

Source	Sum of Squares	df	Mean Square	F-value	P-value	
Model	114.75	9	12.75	109.11	< 0.0001	significant
A (Temperature)	17.52	1	17.52	149.95	< 0.0001	**
B (Mass ratio)	18.03	1	18.03	154.28	< 0.0001	**
C (Stirring speed)	0.12	1	0.12	1.05	0.3400	
AB	0.24	1	0.24	2.05	0.1949	
AC	0.69	1	0.69	5.89	0.0456	*
BC	2.77	1	2.77	23.72	0.0018	**
A^2	39.08	1	39.08	334.45	< 0.0001	**
B^2	14.17	1	14.17	121.22	< 0.0001	**
C^2	14.79	1	14.79	126.56	< 0.0001	**
Residual	0.82	7	0.12			
Lack of Fit	0.52	3	0.17	2.37	0.2113	not significant
Pure Error	0.29	4	0.07			
Cor Total	115.57	16				
R^2	0.993					
Adjusted R^2	0.984					
Adeq Precision	29.05					

Table S2. UPLC-QqQ-MS/MS analytical method validation: The regression equation, precision, accuracy, extraction recoveries, matrix effects and stability of cajaninstilbene acid (CSA), longistyle C (LLC) and 4-*O*-methylpinosylvic acid (MPA) in rat plasma

compounds		CSA			LLC			MPA		
Calibration range (ng/mL)		0.2-570.4			0.2-105.3			0.1-687.4		
Regression equation		y=0.01527x+0.00430			y=0.00119x+0.00034			0.00076x+0.00035		
Correlation coefficient		0.997			0.995			0.998		
LLOQs (ng/mL)		0.2			0.2			0.1		
Spiked conc. (ng/mL)		0.9	22.8	114.1	0.8	21.1	105.3	0.6	13.8	68.8
Measured conc. (ng/mL)		0.9±0.0	22.0±1.0	112.6±3.4	0.9±0.0	20.7±1.3	103.6±3.0	0.6±0.0	12.6±1.1	67.9±2.8
Intra-day	Precision (RSD, %)	4.4	4.6	3.0	3.5	6.3	2.9	5.4	8.9	4.0
	Accuracy (RE, %)	1.5	-3.7	-1.3	3.3	-1.9	-1.6	2.2	-8.5	-1.2
Inter-day	Precision (RSD, %)	5.1	6.4	3.5	3.8	6.8	4.0	5.9	9.1	5.1
	Accuracy (RE, %)	3.0	-2.8	2.1	4.3	1.1	3.0	4.7	-5.3	0.9
Recovery %		85±5	86±5	89±6	85±6	90±6	90±4	86±6	88±7	88±5
Matrix effect %		114±9	108±7	107±9	95±10	102±9	109±79	110±7	90±9	103±10
25 °C for 24 h	Precision (RSD, %)	5.2	4.9	7.3	4.3	5.4	4.9	6.4	4.9	9.2
	Accuracy (RE, %)	3.0	3.0	-5.8	6.3	4.8	-2.0	2.8	2.0	2.3
-20 °C for 30 days	Precision (RSD, %)	6.2	2.5	8.2	5.3	8.2	5.4	3.9	4.8	3.4
	Accuracy (RE, %)	3.5	4.4	1.0	6.4	4.9	3.0	4.4	6.8	3.9
Three freeze-thaw	Precision (RSD, %)	7.2	5.0	6.4	3.2	9.3	7.9	7.3	3.0	10.1
	Accuracy (RE, %)	-2.9	5.5	-0.8	6.4	5.5	3.0	5.2	6.0	3.2
